# The French Medical Service: The Organisation through French Eyes

**Published:** 1915-03-20

**Authors:** 


### THE FRENCH MEDICAL SERVICE.

### The Organisation Through French Eyes.

From a recent number of the *Paris Médical* we take some items of news and some criticisms which, as related to the war, may have some interest for English readers. First there is an announcement that many practitioners driven from Belgium and the North of France are in search of work which will enable them to gain a livelihood, and the journal appeals for information concerning opportunities suitable to this end. The appeal is addressed both to practitioners who have been taken away from their practices by mobilisation and to the mayors of communes where medical help is needed. The number of doctors with the Army is said to be 6,500. Of these ninety-three have been killed, 260 wounded, 440 are recognised as missing, and 507 have been invalided, making a total of 1,300 *hors de combat*. The writer adds that these figures must be remembered when, after the restoration of peace, the tendency will again assert itself to speak with disdain of the doctors as "non-combatants." Proceeding to a general view of the situation, the claim is made that the organisation of the military service is, in certain respects, defective, and that it is wise to recognise these defects, not in a spirit of controversy, but in order that they may be remedied. The statement is made that even yet there are places near the Front where ambulances, though badly needed elsewhere, remain unutilised. On the one hand, too, there are celebrated surgeons who have not seen a single wounded soldier, while certain unhappy physicians, who have never handled a knife, have found themselves confronted with the painful dilemma either of allowing the patient to die for want of intervention, or of undertaking operations for which they are very imperfectly prepared. Telling the story of a small-town which was organised in August to receive 3,000 wounded, the local authorities were faced with the necessity of accommodating 7,000. When, after the most devoted efforts, satisfactory arrangements had been made, the central powers appeared to forget the town; gradually the hospitals were emptied, and the majority had to be closed, though in numerous other places there was great pressure on the accommodation. Suddenly, at the end of January, and without warning, occurs a new development. In the course of a single week 5,000 wounded arrive, and it is necessary to accomplish once again the work of organisation which was undertaken at the beginning and then allowed to lapse for want of opportunity. In August and September such irregularities in the disposal of the wounded might be excused, but since the battle of the Marne it is held they are inexcusable. The absence of great and unexpected events in the fighting ought to have meant the ability to distribute the wounded on a consistent plan, and not sudden alternations from overcrowding at one moment to empty hospitals shortly afterwards. Another criticism is directed against the returns and documents which the medical officer is obliged to furnish, and which, it is said, unduly invade the time necessary for his proper duties. All these and other points have, it seems, been reported to the Ministry, and the writer bases upon this some expectation and hope. At least he notes that a Commission of Inquiry has been appointed, and while he recognises that such a proceeding in time of peace is a convenient method of burying embarrassing questions, he trusts that in time of war a Commission may succeed in arousing the bureaus from their lethargy. As we have all heard stories very similar to this much nearer home, there is, perhaps, some consolation in knowing that our own people are not the only sinners, and that it is not true of everything that they do it better in France. It is right to repeat that the criticisms are all made not only with grace of phrase, but with the obvious desire of securing such improvements as will enable the Service to deal with the heavy demands made on it. Very warm tributes of admiration and gratitude are paid to officers of all ranks and to the various auxiliary workers, who have spent themselves freely and without counting the cost.

In another article, by Professor R. Blanchard, a description is given of the organisation and work of L'Association des Dames Françaises. This was founded in 1876 by Dr. Duchaussoy, and was directed to the training of women in order that they might be of service in military hospitals and ambulances in time of war. It numbers to-day 50,000 members, and has rendered most valuable services to the sick and wounded in all parts of the country. Dr. Duchaussoy, though nearly eighty years of age, continues to direct its operations, and is recognised as one to whom all the sick and wounded, and indeed the country generally, owe a deep debt of gratitude.

Our contemporary announces that a number will appear every fortnight until circumstances permit a return to its usual weekly issue. At the same time it will publish certain special numbers, which will be devoted to matters of military medicine. Dealing with certain domestic questions, the announcement is made that the doctors mobilised at the Front have the right to postpone the payment of their rent, and that others who find themselves in difficulties may gain a similar position on declaration made before certain properly established authorities. The probability is, it would appear, that the Government, after the war, will ask the landlords to grant to tenants a reduction of rent to the extent of from 25 to 30 per cent.

A last point of interest is a series of extracts from letters written by Billroth to his wife in 1870, he then being chief of a military hospital. His frequently repeated counsel is not to believe anything which appears in the newspapers, these being, so he says, more full of lies than ever. Altogether we congratulate our contemporary on an excellent number, produced in circumstances more difficult perhaps than we can readily appreciate.

